# Knowledge of ocular infections among the dental practitioners across India: a cross sectional survey

**DOI:** 10.1186/s13104-023-06656-w

**Published:** 2024-02-01

**Authors:** Ritvi Arvind, Gayatri Bakhshi, Ramya Shenoy, Amit Patil, Ashish Jain, M Roma

**Affiliations:** 1 Manipal College of Dental Sciences, Mangalore, Manipal Academy of Higher Education, Karnataka, Manipal 576104 India; 2 Department of Public Health Dentistry, Mangalore, Manipal Academy of Higher Education, Karnataka, Manipal 576104 India; 3 Department of Conservative Dentistry and Endodontics, Mangalore, Manipal Academy of Higher Education, Karnataka, Manipal 576104 India

**Keywords:** Ocular infestation, Personal protective etiquette, Dental clinics, Protection

## Abstract

**Background:**

Ocular issues such as impaired vision, ophthalmia, orbital cellulitis, and blindness are not common with dental infections. However, there is absence of set guidelines in prevention and management of ocular issues arising from dental infections. Hence the knowledge and vantage point of the dentists with respect to ocular complications arising from dental infections was evaluated.

**Objective:**

This study reviews the knowledge of dentists with association of eye infection due to dental contamination.

**Method:**

A cross sectional survey using standardized questionnaires were sent suing social media platform among the academicians and practicing dental clinicians. All the questionnaires were content validated by three ophthalmologists. Descriptive statistics was scrutinized and tabulated by employing the Statistical Package for Social Sciences (SPSS), version 17 (SPSS Inc., Chicago IL). Chi square tests was used.

**Results:**

About 69.1%(65) and 30.9%( 29) of BDS and MDS grduates have experienced ocular complications because of dental contamination. This distribution showed no statistical significance (p = 0.25). 73.8% (183) and 28.2%( 65) had compliance with the eye protection, 77.7% (160) of BDS graduates and 22.3% (46) of MDS graduates preferred safety eye wear. This distribution showed statistical significance (p = 0.00). About 76.2% (279) of BDS graduates and 23.8%( 67) of MDS graduates faced ocular infections because of different types of splashes which included water, blood and saliva. (p = 0.23)

**Conclusion:**

This study puts an emphasis on the knowledge assessment among the dental practitioners on the importance of preventive barriers, and how special protective gear is required for doing cases undergoing dental treatment.

**Supplementary Information:**

The online version contains supplementary material available at 10.1186/s13104-023-06656-w.

## Introduction

Each profession has its own merits and demerits, and protecting one’s well-being is of prime importance. Ocular manifestations are always highly risked with dental occupation, but however one can avoid it by following proper personal protective equipment etiquettes. Over the past few years with COVID-19 scenario, incredible attention and increased awareness has uprooted the outook among the dentists and patients for the absolute need on personal safety and infection control measures to prevent cross contamination. Besides, there should be adequate knowledge about eye infection and protection and the measures to prevent and mange it.

Occupational eye injuries emerging due to mechanical, microbiological, and chemical insults results in dysfunctional ability to work with ease among the dental personnel [1, 2]. The dental fraternity are highly risked among the health care workers towards the eye injuries. In an article by Kandyce, et al. it was predicated that the 6% of the occupational injuries related to dentistry are eye related [3]. Eye injuries may have deleterious effects. The signs and symptoms are often related to the type and the impact of the injury. It can be due to mechanical trauma, aerosol vapors, presence of spicules of tooth or decay arose during the tooth preparation or cavity preparation. The effects can range from mild effects like presence of sensation of foreign body in the eye, blurring, watery eyes, increased lacrimation, and yellowish discharge to severe effects like perforation and blindness (1). These occupation induced eye injuries may have significant consequences and may result in scleral and retinal damage. The dentist ought to protect his or her eyes from the various chemical and mechanical insults with protective goggles with side shields to prevent life-long effects on the eyes. Centre for Disease Control and Prevention (CDC) has postulated that protective glasses should be mandated for both the patients and dentists to prevent cross contamination [2]. British Dental Association charted, “Infection Control in Dentistry” which highlighted the need for protective eye wear for the dental personnel (dentist and assistant) and patients [3].

Dental induced ocular contamination with body fluids like saliva and blood can produce heinous effects. These contaminations can be an assembly of various micro-organisms like bacteria, virus, fungi, etc. causing blepharitis to keratitis [2, 4]. Literature review search has put forward that wearing contact lens during dental procedures can result in protozoal infections which can be harmful [5]. Exposure to blood and body fluids play a major role in eye infections in dentists [4]. The eye infections among the dentists were attributed to insufficient protection to the eyes [1]. In spite of escalated vulnerability to systemic problems, eye care practices by dental fraternity following traumatic exposure to aerosol and splashes is given less importance and there is no research- based confirmation protocols on the same [4, 6, 7]. Studies have shown that eye injuries among the dentist’s occur due to deficient protection to the eyes during the treatment procedure [4, 8]. It is also proven that dentist’s are being non-compliant with eye wear protection and prefer to use customary eye glasses which don’t yield full protection [4, 9].

All the dental procedures like scaling, excavations, etc. are accomplished with rotary handpieces [9, 10]. Variety of impurities like spurs of decay, restorative materials, blood products etc. are liberated which get clogged into tissues. The aerosols generated from the rotary handpiece is an assemblage of microbes causing infections [9, 11].

Dental medicine is undoubtedly a parlous profession for eye infections on a regular basis. Array of microorganisms (bacteria, protozoal, viruses, fungi) can lead to ocular infestations ranging from slight eyelid swelling to total blindness [12, 13].

Protective eye wear during dental procedures prevent the splashes and spicules from entering the eye [12]. Literature has stated that knowledge of eye protection among dental personnel is lacking which can be the perilous source of ocular injuries [14, 15]. A lot of data has not been emphasized on the importance of the eye wear among dental fraternity. Absence of adequate qualification, noncompliance with safety glasses and unable to work with them are bound to lead to ocular incidents. Assertiveness about infection-control policies, and ocular safety practices should be mandated among dental clinicians in their years of education and proper implementation has to be set in their working practice [16].

This study puts an emphasis on the knowledge assessment among the dental practitioners on the significance of personal protection equipment for doing cases undergoing dental treatment.

## Main text

### Materials and methods

A pre-designed questionnaire based on literature was developed by two authors [2]. The content and construct validation was carried out by two opthomologists and changes were incorporated. External validation was done by administrating the questinnaore to two BDS and two MDS graduates.The validated, self-reported questionnaire was circulated by means of various social media platforms like Whatsapp, Facebook, and Instagram among dental personnel [https://forms.gle/5QvKFxkLabRzasHD7]. The objective of the study was to analyze the knowledge among dental practitioners across India in relation to contracting ocular infections upon doing dental procedures and on using personal protection barriers for prevention of the same. The cross sectional survey included demographics, awareness of eye infections owing to dental contamination, it’s responses, and preventive strategies. The content and construct validity of questionnaire was done by three ophthalmologists. This study was approved by the Institutional Ethical Committee of the University bearing the IEC number 20,049. 400 registered dentists and academicians responded to the questionnaires. The target audience included all the dentists working in government and private sectors and as academics. Anonymity was maintained during the course of the study.

### Statistical analysis

Descriptive data was tabulated using Statistical Package for Social Sciences (SPSS), version 17 (SPSS Inc., Chicago IL) and Pearson Chi square tests were used.

## Results

The responses were recorded among the dental practitioners and were grouped according to their degree of education levels.

Knowledge of ocular complications among dentists with under graduate qualifications.

69.1% of surveyed respondents knew and had experienced the ocular infections because of dental contamination. 79.7% weren’t aware about such problems. Among the respondents who were aware about the ocular infections because of dental contamination, 77.2% stated net importance of eye protection [Table 1]. 81.2% preferred powered glasses for protection and 77.7% agreed on safety eye wear glasses being the best for eye protection [Table 2]. 76.2% dentists faced ocular infections because of different types of splashes which included water, blood and saliva [Table 3]. Hence concluding that ocular infections were faced by the significant number of the respondents and are in the favor of wearing eye protection as the basic protection against all the ocular complications taken place during any dental procedure.

**Table 1:** Ocular complication experienced as per the speciality of work

			Experienced ocular complication		Total
			YES	NO	
Speciality of work	BDS	Count	65 (69.1%)	244 (79.7%)	309
	MDS	Count	29 (30.9%)	62(20.3%)	91

Pearson Chi-Square 4.58 ; df value 1 ; *P* value = 0.25

**Table 2:** Type of eye protection based on specialty of work *

		Type of eye protection			
			NONE	SAFETY EYE WEAR	POWERED GLASSES
Speciality of work	BDS	40(90.9%)	4(100%)	160(77.7%)	95(81.2%)
	MDS	4(9.1%)	0(0.0%)	46(22.3%)	22(18.8%)

Pearson Chi-Square 38.263a; df value 6; *P* value = 0.00

**Table 3:** Type of aerosol or splash experienced among BDS Vs MDS

		Type of splash				All the Above
		NONE	SALIVA	BLOOD	WATER	
Speciality of work	BDS	3(75%)	11(100%)	4(66.7%)	12(92.3%)	279(76.2%)
	MDS	1(25%)	0(0.0%)	2(33.3%)	1(7.7%)	87(23.8%)

Pearson Chi-Square 5.52; df value 4; *P* value = 0.23

Knowledge of ocular complications among dentists with post graduate qualifications.

30.9% of surveyed respondents knew and had experienced the ocular infections because of dental contamination. 20.3% weren’t aware about such complications [Table 1]. Among the respondents who were aware about ocular infections because of dental contamination, 26.2% stated net importance of eye protection [Table 4]. 66.7% preferred spectacles for protection whereas 75.0% agreed on Loupes being the best for eye protection [Table 2]. 23.8% dentists faced ocular infections because of different types of splashes which included water, blood and saliva [Table 3]. Hence concluding that ocular infections were faced by the significant number of the respondents and are in the favor of wearing eye protection as the basic protection against all the ocular complications taken place during any dental procedure.

**Table 4:** Compliance with eye protection based on specialty of work

		Compliance with eye protection			
		NOT IMPORTANT	ALL THE TIME	MOST OF THE TIME	PART TIME
Speciality of work	BDS	0(0%)	183(73.8%)	99(81.1%)	27(93.1%)
	MDS	1(100%)	65(26.2%)	23(18.9%)	2(6.9%)

Pearson Chi-Square 10.28; df value 3; *P* value = 0 0.016

## Discussion

It is of prime importance to have extensive information of the eye infections caused during dental procedures and also evaluation of dentist’s understanding and their assertiveness towards helping in expanding the preventive techniques towards ocular infections. Due to development of infection into the orbit can lead to other complications arising through an expansion of pathways [17].

After the assessment, the extent of expertise and frame of mind of the dentists toward ocular infections owing to poor protective eye wear has been surfaced many times. The observations highlighted the level of knowledge about ocular signs and symptoms because of dental contamination during dental procedures is inadequate among dentists.

From the present data, approximately 27.5% respondents did not understand that ocular infections occurring because of poor eye wear protection. It is important for dentists to be absolutely geared up with modern day statistics, remedy modalities, and knowledge of assets available on a day by day foundation.

In this document, 48.5% answered that they accumulated the knowledge from textbooks. 51% read extensively on the internet about ocular infections due to personnel experience and 19.7% read about the complications and have the knowledge through journals. Nowadays, numerous varieties of mass media were determined that may be efficaciously used as informatory supply. Our data highlighted that net and online journals were crucial sources of information.

Although research and proper statistics via textbooks, regarding the preventive measures of ocular infestations has been stated, however only few of the contributors (graduates and postgraduates) liked the weightage of ocular infections. Approximately 26.8% dentists stated that ocular complications aren’t extreme in nature. However major portion of dental fraternity (graduates and postgraduates) have been unaware of the price of seriousness of ocular complications. Since eye complications are temporary and uncommon to occur but there can be instances of permanent visible loss if the problem is ignored.

The solemnity regarding ocular signs and symptoms is of less importance to the dentist due to the perception that eye manifestations are very uncommon and remaining for brief duration. The prevalence rates of the ocular injuries owing to dental infections have reported to be low, and hence the clinician’s attentiveness to ocular injuries has been considered of less significance. Various authors have reported their experience of ocular manifestations due to dental infections in presenting case reports [18–24]. Hence, the clinician should consider the impact of ocular manifestations and should strive to fend off the infections at the primary level.

However, the dental curricula fail to mention the preventive approaches and preliminary treatment of eye manifestations as a result of dental contamination. It is considered as the need of the hour to optimally formulate tips for the dentists to avert ocular complications and additionally to expand the awareness and assertiveness on diagnosing, coping and managing preliminary ocular infections. Therefore, right recommendations need to be formulated for the precautionary measure of the ocular manifestations [25, 26].

Furthermore, new trend of education for the dentist must be promoted with specialized training via continual dental training, workshops, and lectures to foster preliminary protection of ocular injuries due to dental contamination in clinical setup and aid in prompt remedial sessions with ophthalmologist [16, 17, 26].

The deficiency in research related to ocular injuries owing to dental induced contamination must be justified by furnishing ample literature and statistics via social media, articles, journals, textbooks, continuing dental education programs, workshops, and infection control training programs and should be included in the dental curriculum [14, 17]. Educating the dental professionals regarding the importance of eye infections occurring due to various operative, restorative and other dental procedures and strategic approach of prevention and management should be mandated.

## Conclusion

This study delineates that our data has discovered low degree of information about ocular infections because of dental contamination amongst dental surgeons in India. The comprehension towards the problem is adequate but wishes in addition of improvement. Addition of proper protocol for prevention and preliminary care of eye infections caused owing to dental contamination should be included in the curricula of dentistry. Furthermore, extra facts need to be made accessible to dentists via studies, continual dental schooling, workshops, and symposiums.

### Electronic supplementary material

Below is the link to the electronic supplementary material.

Supplementary Material 1

## Data Availability

The datasets used and/or analyzed in this study are available from the corresponding author upon reasonable request.

## Abbreviations

SPSS: Statistical Package for Social Sciences
COVID-19: Coronavirus Disease − 19
CDC: Centre for Disease Control and Prevention
