# Supplementary material for: Knowledge of ocular infections among the dental practitioners across India: a cross sectional survey
Source: BMC Res Notes. 2024 Feb 1;17:41. doi: 10.1186/s13104-023-06656-w (PMC10836011; doi:10.1186/s13104-023-06656-w)
Supplement: Supplementary file 1 — Supplementary Material 1 [file 13104_2023_6656_MOESM1_ESM.docx]

**Questionnaire (**More than one option can be selected)

1. Choose your specialty of work:

Oral & Maxillofacial surgeon

Prosthodontist

Periodontist

Orthodontist

Oral Physician & Maxillofacial Radiologist

Oral Pathologist

Paedodontist

Endodontist

Public Health Dentist

General Dentist

2. Years of experience (after highest qualification achieved):

Less than 5 years

5-10 years

11-15 years

More than 15 years

3. Are you aware of the ocular complications due to dental infection?

Yes

No

Don’t know

If yes, please mention the knowledge on source of information?

Personal experience

Textbook

Internet

Journals

Case report articles

All of the above

4. Have you come across any ocular complication in yourself due to dental procedure?

Yes

No

5. What type of symptoms did you experience?

Redness and Itchy eyes

Increased lacrimation

Watery discharge from the eyes

Defective vision

Eye inflammation

Eye flu

Contraction of the visual field

Loss of vision

6. a) How long did the symptoms last?

Few seconds

Few minutes to few hours

Few days

Few months

6 b) Did you consult an ophthalmologist for the ocular complications?

Yes

No

7. According to you, what could be the source of ocular complication due to dental procedure?

Bacterial

Viral

Aerosol generated procedure

Chemical splash in the eye

Irritating substance or material in the eye

All of the above

8. Compliance with eye protection

a) According to you, is the eye protection protocol important?

Never important

Part of the time

Most of the time

All the time

b) What type of eye protection do you follow?

Safety eye glasses

Powered eye glasses

Visors

Loupes

c) Please tick the boxes for the procedures in which you use eye protection?

Oral examination

Oral prophylaxis

Orthodontic treatment

Root canal treatment

Tooth preparation

Cavity preparation

Extraction and suturing

Irrigation

Root planing and curettage

Laboratory assisted procedures like denture trimming, polishing, etc

Manipulation of dental materials like cements and impression materials

Surgical procedure

Biopsy

9. According to you what type of foreign body could be the cause for occupational exposure to eye trauma?

Tooth particle

Dental material particle

Carious particle

10. What type of splash be the reason for occupational exposure to eye trauma?

Saliva

Blood

Water

Mix

11. What type of post exposure practices may be followed by dental practitioners after exposure splash from the patient?

Stopping the treatment procedure and rinsing of eyes under running water

Use of tissue to clean eye

Rubbing of eye with the back of gloved hand

Visit to the ophthalmologist

Use of eye drops
